# Tailoring Educational Materials to Cultural Context Matters

**DOI:** 10.2196/42604

**Published:** 2023-11-06

**Authors:** Sasha Zaki

**Affiliations:** 1 Jinnah Sindh Medical University Karachi Pakistan

**Keywords:** Bangladesh, health education, health knowledge, quality of life, motivation, randomized controlled trial, RCT, campaign, chronic kidney disease, knowledge, mobile health, mHealth, kidney, chronic disease, chronic condition, patient education, patient knowledge, low- and middle-income countries, LMIC

I have read with immense interest the article published by Sarker and colleagues [1] titled “Chronic Kidney Disease Awareness Campaign and Mobile Health Education to Improve Knowledge, Quality of Life, and Motivation for a Healthy Lifestyle Among Patients With Chronic Kidney Disease in Bangladesh: Randomized Controlled Trial.” The authors detailed how health education campaigns and interventions can facilitate chronic kidney disease (CKD) awareness among patients and help them maintain health-related parameters such as fasting blood sugar levels and blood pressure (BP) [1]. All in all, this was an insightful read, and the authors are to be commended for designing such a meticulous study. However, the authors are advised to comment on a few concerns.

First, the community health workers conducted physical examinations to measure characteristics such as BP and waist and hip circumferences. However, it has not been explained how these measurements were taken. In another study, BP was recorded via a calibrated digital automatic monitor while hip circumference was measured at the maximum prominence of the buttocks [2]. Explaining how the participants’ baseline and subsequent follow-ups were measured helps to standardize the procedure undertaken and to establish the reproducibility of the study findings.

During the campaign, participants of the intervention group were given CKD textbooks and leaflets [3]. These educational materials were compiled by a diverse research team based on the content available on the National Kidney Foundation website. Although the content was written in Bangla (the native language), it seems as though no other measures were taken to ensure that the material was relevant to the Bangladesh context. Additionally, when compared with other features of the campaign—particularly the nephrologist-facilitated lecture and discussion as well as the mobile education—it is unclear whether the books and the leaflets played a significant role in enhancing patients’ education and awareness. The effectiveness of the educational material becomes even more dubious given that almost half of the participants of the intervention group had no formal education.

Moreover, interviews took place at baseline, 3 months, and 6 months. This yielded qualitative data; however, it is unclear how or if these data were processed. These interviews could have shown changes in patients’ perception of CKD, improved awareness about this disease, how their quality of life enhanced as well as other preventative measures undertaken by the participants (eg, getting adequate sleep) that showed motivation for a healthy lifestyle. This would have supplemented the data measured in this study.

## Abbreviations

BP: blood pressure
CKD: chronic kidney disease
